# Nano-pulse stimulation™ therapy (NPS™) is superior to cryoablation in clearing murine melanoma tumors

**DOI:** 10.3389/fonc.2022.948472

**Published:** 2023-02-08

**Authors:** Amanda McDaniel, Bruce Freimark, Cebrina Navarro, Kristin Von Rothstein, Dacia Gonzalez, Keith Linder, Richard Nuccitelli

**Affiliations:** ^1^ Department of Biology, Pulse Biosciences, Hayward, CA, United States; ^2^ Department of Dermatopathology, Linder Pathology Services, Raleigh, NC, United States

**Keywords:** nano-pulse stimulation therapy (NPS), regulated cell death, cryoablation, B16-F10, melanoma, dermal fibrosis and scarring, nanosecond pulsed electric fields (nsPEF)

## Abstract

**Background:**

Nano-Pulse Stimulation™ Therapy (NPS™) is a new, bioelectric modality that applies ultrashort pulses of electric energy to trigger regulated cell death in treated tissues. Instead of initiating necrosis by heating or freezing, NPS therapy permeabilizes intracellular organelles to activate the cell’s own self-destruct pathway of programmed or regulated cell death. Unlike cryotherapies that can both damage structural tissues and diffuse into the periphery beyond the margins of the lesion, NPS only affects cells within the treated zone leaving surrounding tissue and acellular components unaffected.

**Methods:**

We generated melanoma tumors in mice by injecting B16-F10 cells intradermally and compared the efficacy and resulting skin damage from Nano-Pulse Stimulation Therapy with that of cryoablation in clearing these tumors.

**Results:**

The results of the study demonstrate that NPS is superior at clearing B16-F10 melanoma lesions. NPS permanently eliminated up to 91% of all tumor lesions with a single treatment compared to cryoablation that only eliminated up to 66%. Importantly, NPS permanently eliminated these lesions with no recurrence and with minimal dermal fibrosis, underlying muscle atrophy, permanent hair follicle loss or other markers of permanent skin damage.

**Conclusions:**

These findings suggest that NPS is a promising new modality for the clearance of melanoma tumors and is a more efficacious, less damaging approach than cryoablative methods for the treatment of aggressive malignant tumors.

## Introduction

Cutaneous melanoma is the most aggressive and lethal form of skin cancer. Over the past few decades, the incidence of melanoma has been steadily increasing and in 2022 alone there are projected to be nearly 100,000 new cases diagnosed, with over 7% of those cases resulting in mortality (1). Early-stage disease is usually managed with surgical excision alone, but removal of the malignant tissue eliminates further exposure to the immune system. Some other methods to ablate lesions have the additional advantage of initiating an immune response. Two such therapies are cryotherapy (2) and Nano-Pulse Stimulation Therapy (3) so both have been used in this study to compare the efficacy and skin damage resulting from each of them.

### Nano-pulse stimulation therapy

Every cell in our bodies contains a fail-safe mechanism called regulated or programmed cell death that allows it to self-destruct when it reaches the end of its useful life, encounters a lethal gene mutation or an injury that it is unable to repair (4–6). Nano-Pulse Stimulation™ Therapy (NPS™) activates this pathway using ultrashort electric pulses. Unlike direct-contact ablation technologies that kill cells by necrosis using heat or cold, NPS is a bioelectric energy modality that triggers the cell’s natural self-destruct pathway by initiating a transient permeabilization of the plasma and organelle membranes of targeted cells without causing thermal damage. This alters the function of internal cellular organelles, including the mitochondria and endoplasmic reticulum (7), without disrupting the extracellular tissue, primarily collagen-rich dermal foundation. The current lesion size limitation is 1 cm in diameter for a single treatment, but larger lesions can be treated with multiple applications. Previous published work includes treatments of seborrheic keratosis (8), sebaceous hyperplasia (9), warts (10) and basal cell carcinoma (11). In animal studies, NPS has shown high efficacy in treating a variety of malignant murine tumor types including rat hepatocellular as well as mouse breast, fibrosarcoma, squamous cell carcinoma (SCC), pancreatic, lung and melanoma tumors (12–18).

### Cryotherapy

Due to the low cost of cryoablation, it has become an alternative to other more traditional surgical methods for cancer treatment. Cryoablation has been used to treat bone, cervical, eye, kidney, liver, lung, and prostate cancers (19–22). Some of the noted drawbacks to cryoablation have been the potential for scarring and long-term nerve damage caused by the treatment itself (23), as well as a question as to its long-term efficacy and ability to prevent microscopic spread of cancers (24, 25). While cryoablation and NPS are both considered focal therapies, NPS only affects cells between the two sets of microneedles of the applicator while cryoablation spreads beyond the applicator surface due to thermal diffusion.

In this study we demonstrate that NPS has superior efficacy in clearing a B16-F10 murine melanoma tumor, without reoccurrence with minimal dermal fibrosis and tissue damage. Since NPS is highly efficacious while producing less damage to tissue it is a promising minimally invasive physical modality for the treatment of tumors, particularly those that are not surgically resectable.

## Materials and methods

### *In Vivo* tumor model

Mice: Female C57BL/6J mice, 6-8 weeks old (Jackson Laboratories, Sacramento, CA) were acclimated for at least 3 days before treatment, housed in groups of 10, and both flanks were shaved before the start of tumor inoculations. Temperature and humidity were monitored daily, and animals were maintained on a 12-hour light/dark cycle. Water (Milli-Q) and food (Pirolab Diet 20 chow) were given *ad libitum*. All experiments were performed in accordance with animal care guidelines set forth by the Pulse Biosciences IACUC.

Tumors: The B16-F10 tumor line was obtained from ATCC (Manasus, VA, cat # CRL-2539) and propagated in tissue culture with DMEM supplemented with 10% v/v fetal bovine serum (FBS), penicillin/streptomycin, and harvested for inoculation between passages 9-12 for all studies. Tumors were initiated in mice by intradermal (i.d.) injection into the right flank with 2x10^5^ cells/30µL in Hank’s balanced salt solution (HBSS). Tumor growth was measured twice a week by calipers. The volumes were determined using the formula: volume = length x width^2^/2. Tumors were randomized to treatment groups when the largest diameter reached ~5mm on Day 6 post tumor inoculation (PTI). Mice were removed from the study if the animal lost more than 20% of their initial body weight, appeared moribund, the tumor was ulcerated, or the tumor volume exceeded 2000mm^3^. The day at which each mouse was sacrificed or found dead was recorded and used to generate a Kaplan-Meier survival curve. Mice who cleared the lesion were continually monitored for tumor regrowth until study completion on Day 65, at which point mice were sacrificed and skin lesions removed for histological analysis.

### NPS and cryoablation tumor treatments

**Figure 1:**
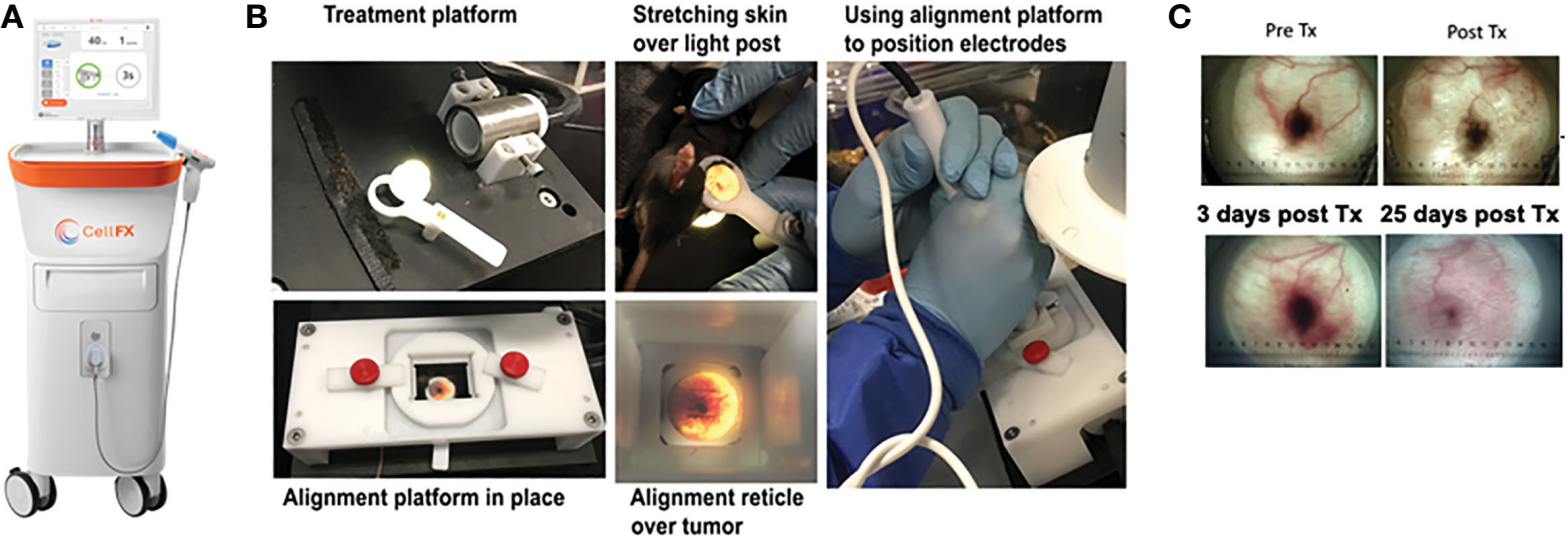
Images of the CellFX™ treatment platform and a treated melanoma tumor. **(A)** CellFX Pulse Generator; **(B)** Montage of images illustrating the procedure used to treat the melanoma tumors by stretching the skin containing the tumor over a translucent silicone light post and aligning the tumor with the application electrode followed by treatment with the CellFX system; **(C)** Transillumination images of a typical melanoma over time, Before treatment, immediately after treatment, 3 days post treatment and 25 days after treatment.

Six days post-inoculation, mice that developed tumors were treated with either NPS energy delivered by the CellFX^®^ System (Pulse Biosciences, Hayward, CA) or cryotherapy using a 5mm closed-end conical metal cryoprobe (Brymill, Ellington CT, model CRY-AC-3 B800). Tumors were injected into the intradermal space within the skin so that they could be stretched over a platform designed to isolate them from the body and internal organs (**Figure 1**). Before treatment, each cage of mice was placed into a chamber containing 2.1% isoflurane in oxygen to induce an anesthetized state. Once mice were recumbent, each mouse was individually placed onto the treatment platform, receiving inhaled isoflurane directly from a nose cone for the duration of the procedure, typically 2-3 minutes. Upon completion of each treatment mice were returned to their home cage for recovery. All procedures were performed according to IACUC-approved protocols.

### Nano-pulse stimulation (NPS) therapy

**Figure 2:**
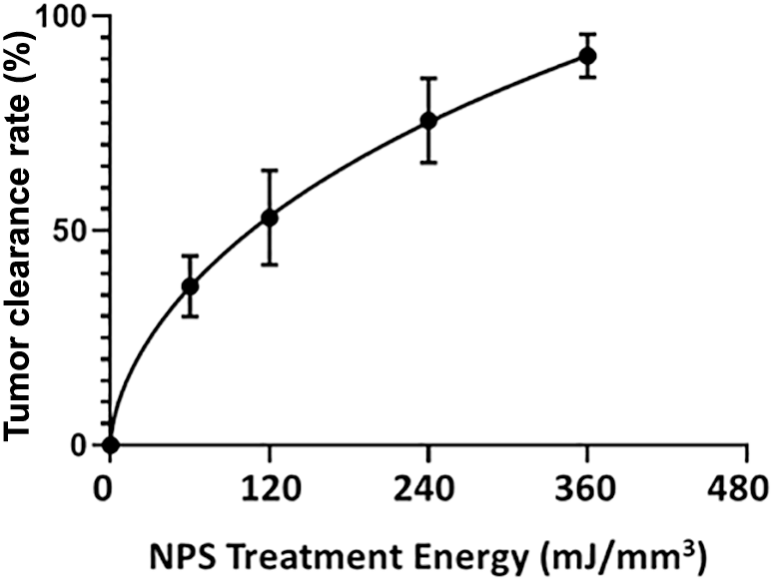
The percentage of treated tumors that are completely cleared as a function of the energy applied during treatment. Bars represent the Standard Error of the Mean.

NPS therapy was delivered using a 5.0 × 5.0 × 3.5mm treatment tip attached to a handpiece plugged directly into the CellFX^®^ device. The treatment tip contained two rows of 5 microneedles 3.5 mm long, spaced 5mm apart. Mouse tumors were treated by stretching the skin containing the tumor over a translucent silicone treatment post and inserting the probe needles to flank the sides of the tumor (**Figure 1**). A light source housed under the treatment post was employed to illuminate the tumor treatment area to aid in placement of the microneedles around the tumor. Each tumor received either a low-mid dose of 180 mJ/mm^3^ or a high dose of 360 mJ/mm^3^. The energy doses chosen were selected based on previously performed dose-response tumor clearance studies (**Figure 2**). The low-mid dose was established as effective at clearing >60-70% of all treated tumors and the second higher dose was capable of clearing >90-100%.

### Cryotherapy

Cryotherapy was delivered as a single dose of a cryosurgical system with a 5mm closed-end conical probe cooled with liquid nitrogen applied directly to the tumor (Brymill Corp., Ellington CT). The cryoablation dispenser (model CRY-AC-3 B800) was filled with liquid nitrogen and the probe tip was pre-chilled to -40°C as measured by a thermal imaging camera (FLIR, Estonia; model FLIR-E64501). Mouse skin was stretched across the treatment post and cryotherapy was applied to the tumor for the designated time, during which the temperature was continually monitored, and the probe received a cooling burst every ten seconds to keep the temperature stable at -40°C. Cryoablative temperatures rely on the formation of ice crystals in tissues as the cell death mechanism (26). Durations of exposure, defined as “doses,” were chosen based upon previous clinical findings showing that exposures under one minute were less likely to induce complete cell death of all tumor cells, than were exposures lasting longer. Exposure length is thus a critical variable as longer exposures are more likely to permanently eliminate a lesion (21, 27). We chose 45 seconds (45s) as the low-mid dose and 90 seconds (90s) as the high dose.

### Skin biopsies

After euthanasia, a rectangle of skin (2.5 cm long by 1.5 cm wide) containing the treatment area in the center, was excised andattached flat to paper card stock without stretching, and submersed in 10% neutral-buffered formalin. After 24-48 hours of fixation, each skin sample was bisected in the center of the treatment area, samples were marked with surgical ink to maintain orientation, and both halves were embedded in paraffin along their treatment area cut surfaces. Samples were routinely processed for paraffin histology (AcePix, Hayward, CA), sectioned to 5 micrometers, and stained with hematoxylin and eosin (H&E) or Gomori’s trichrome.

### Histological analysis of skin samples

Assessment of histopathology was performed by a board-certified veterinary pathologist with expertise in dermatopathology. Skin treatment areas were compared for treatment-related tissue scarring and injury that included dermal fibrosis, width of fibrosis, hair follicle loss, intactness of epidermis, cutaneous trunci muscle atrophy/loss, and inflammation. Dermal fibrosis was identified by linearization, compactness, and thickness of dermal stroma. Cutaneous trunci muscle atrophy/loss was recognized as segmental muscle thinning or absence in the treatment area. Skin lesions were scored using a standard severity scale: 0 = no change, 1 = mild, 2 = moderate, and 3 = marked (**Table 1**). All histology slides were randomized and scored in a blinded manner. If lesions differed in sections from the same treatment, then the most severe lesion was scored. Histopathology scores were compared across all groups using a Kruskal-Wallis one-way analysis of variance (ANOVA).

**Table 1:** Severity scoring of markers of tissue damage.

Marker	Definition	Scoring System
**Dermal Fibrosis**	Linearization and compactness of dermal collagen and thickness of dermis	0=no lesion, 1=mild, 2=moderate, 3-marked
**Lesion size**	Width of dermal fibrosis was scored	0=no lesion, 1- mild, 2=moderate, 3=marked
**Hair follicle loss**	Number of follicles missing in area of fibrosis	0=no follicle loss, 1=mild, 2=moderate, 3=marked
**Muscle Atrophy/loss**	Thinning of panniculus (twitch) muscle, thinning of muscle fibers and loss of muscle fibers	1=Partial loss (thinning), 2=Full-thickness loss of muscle for short distance 5-6 follicles wide or less; 3=Full-thickness loss of muscle greater than 5-6 follicles
**Inflammation**	Amount of inflammation	0=no inflammation; 1=mild, 2=moderate, 3=marked

### Statistical analysis

**Figure 3:**
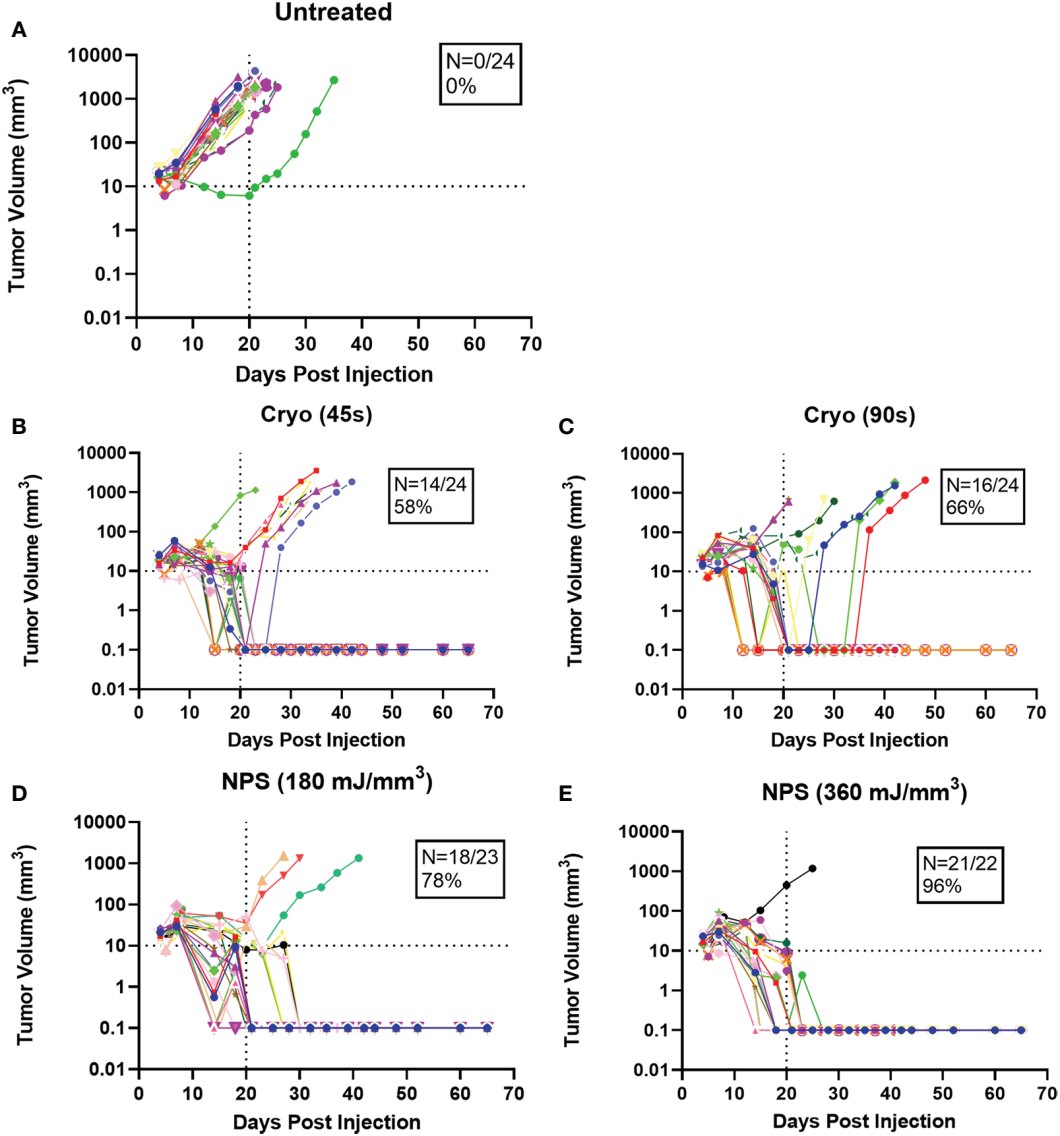
Growth rates of individual tumors treated with the treatment indicated above each graph. Each color represents a single tumor. N represents the percentage of tumors completely cleared in each case **(A)** Untreated; **(B)** Cryo (45s); **(C)** Cryo (90s); **(D)** NPS (180 mJ/mm3); **(E)** NPS (360 mJ/mm3).

**Figure 4:**
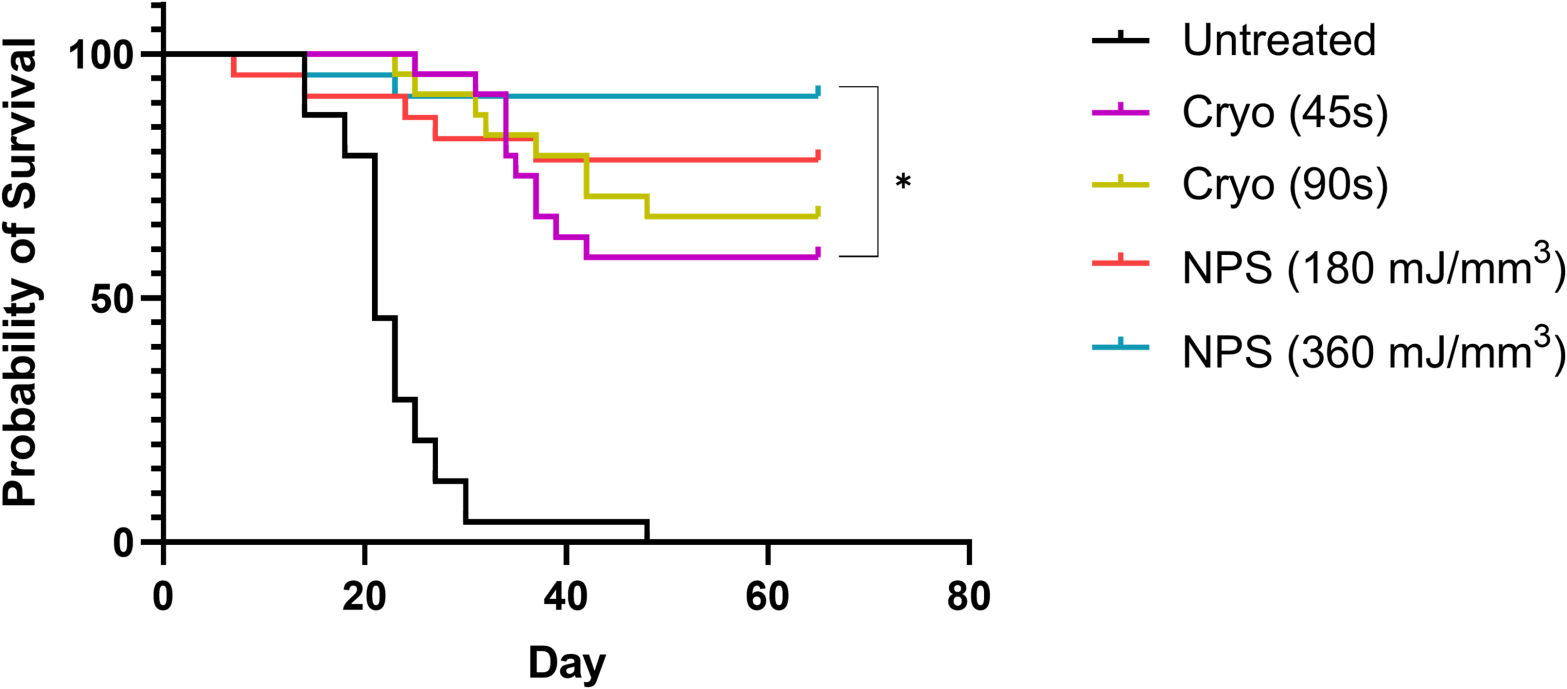
Kaplan-Meier Survival data to day 65 for each treatment. The high dose NPS group survival is significantly better than the 45s cryo treatment group. Both treatment does of NPS had higher rates of survival than either cryo group (log-Rank Mantel-Cox test, *p<0.05).

Statistical analyses were performed using GraphPad Prism software (v9, La Jolla, CA). Tumor elimination rates were compared between groups using a chi-square contingency test (**Figure 3**). Kaplan-Meier survival curves were compared by log-rank (Mantel-Cox) test (**Figure 4**). Kruskal-Wallis non-parametric ANOVA was used to compare histopathological scores generated for each marker of tissue damage for histological samples (**Figure 4**). A two-tailed p-value of < 0.05 was considered statistically significant (*p<0.05; **p<0.01; ***p<0.001).

## Results

### Efficacy of tumor clearance

**Figure 5:**
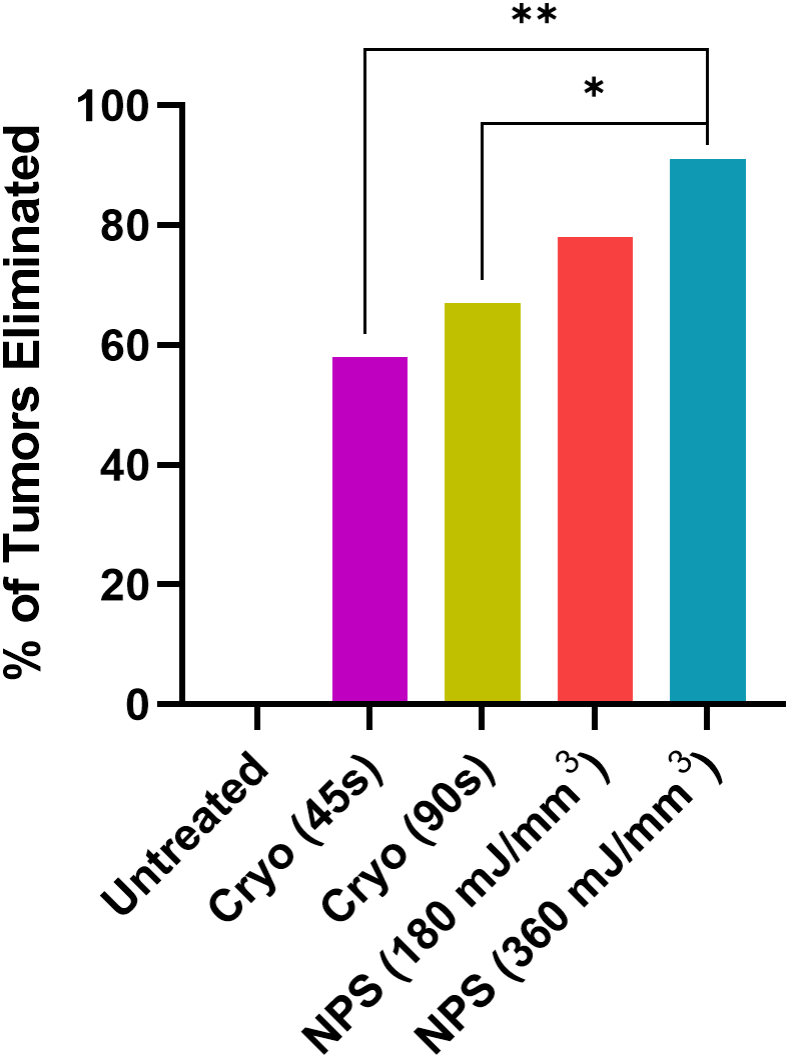
The percentage of tumors that were completely eliminated by the indicated treatments. Both low-mid (180 mJ/mm^3^) and high (360 mJ/mm^3^) NPS treatment groups exhibited higher rates of complete tumor elimination than either the low (45 s) or high (90 s) cryoablation treatment groups. (Chi square test: *p<0.05, **p<0.01).

Intradermal B16-F10 murine melanoma tumors grow rapidly in mice and normally reach a size that requires euthanasia within 3 weeks (**Figure 3A**). However, treatments with both NPS and cryoablation greatly slow this growth and usually result in tumor shrinkage within 2 weeks. Tumors that cleared following NPS treatment remained cleared and did not recur. However, even after initial clearance with cryoablation, tumor growth would resume for many of the tumors within 20-30 days of initial clearance. When mice were treated with a low-mid dose (180 mJ/mm^3^) of NPS, tumors were permanently eliminated in 78% of mice (18/23) and when treated with the higher dose (360 mJ/mm^3^) of NPS energy the percentage increased to 96% (21/22). In contrast, the lower dose of cryo (45s) eliminated only 58% (14/24) of all tumors and the higher dose (90s) only showed a slight improvement to 66% (16/24). The higher dose of cryo exposure failed to reach the level of efficacy of even the low-mid dose NPS. The rate of complete tumor elimination was significantly greater in the high dose NPS (360 mJ/mm^3^) group compared to both the low-mid (**p=0.0096) and high (*p=0.0391) dose cryo groups (**Figure 5**). The efficacy of each treatment group was also reflected in the survival rate. Mice treated with high-energy NPS (360 mJ/mm^3^) were the most likely to survive until the study endpoint (91%) and this was significantly longer than for mice treated with a low dose of cryo (58%) (**Figure 4**; *p=0.0159).

### Histological analysis of post-treatment tissue

**Figure 6:**
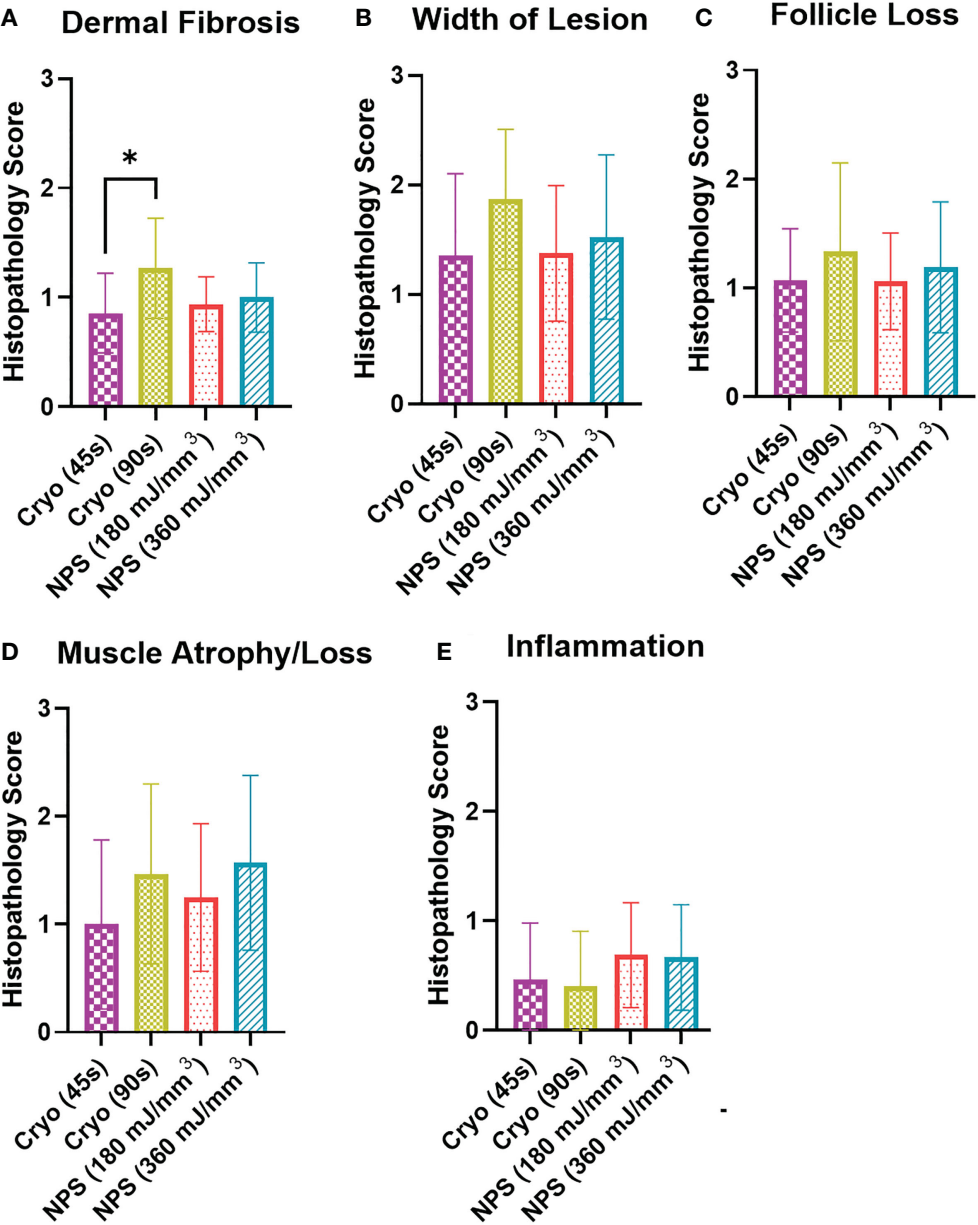
Histopathology scoring of histology sections collected on day 65 from each tumor treatment. Scale: 0=no lesion, 1=mild, 2=moderate, 3= marked. **(A)** Dermal fibrosis was significantly higher for the 90s cryo treatment than the 45s treatment (Kruskal-Wallis ANOVA, *p<0.05); **(B)** Lesion width showed no significant differences between the different treatments; **(C)** Hair follicle loss was similar for all treatments; **(D)** Muscle atrophy/loss: 1=partial loss; 2= full-thickness loss of muscle for short distance; 3=extension of atrophy beyond full-thickness loss. There was no significant difference in atrophy among the four treatments; **(E)** Inflammation score indicated only very minor inflammation at 65 days for the four treatments.

**Figure 7:**
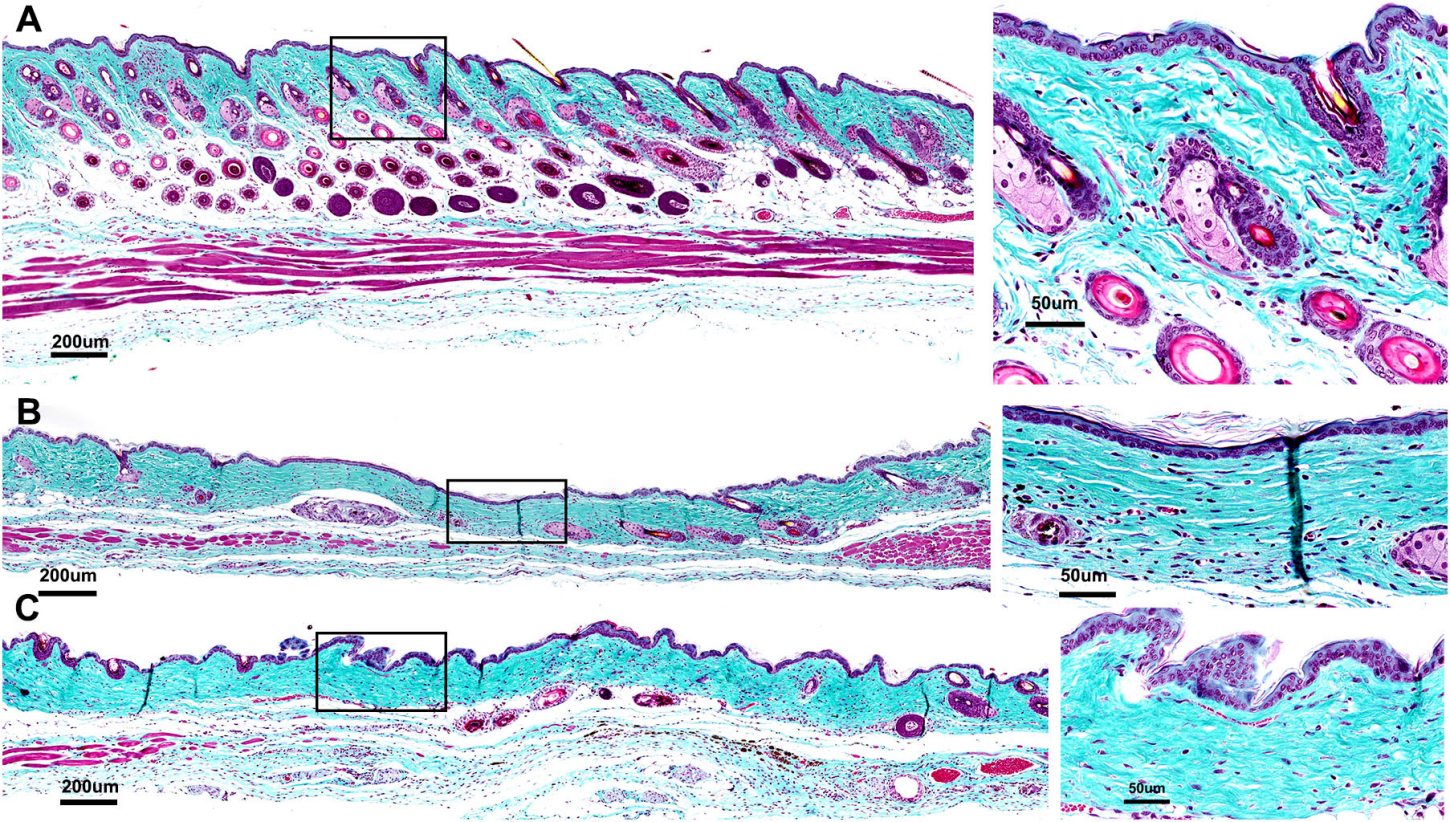
Histological sections of skin regions where the tumor had been treated stained with Gomori’s trichrome to assess collagen linearization and compaction (inset 4X greater magnification of black box region marked on the left). **(A)** Section showing no damage in which collagen and muscle are structurally intact with no loss of hair follicles; **(B)** Mild-moderate damage indicated by slight linearization and compaction of collagen indicating dermal fibrosis and loss of hair follicles; **(C)** Moderate-Marked damage indicated by moderate linearization and compaction of dermal collagen, clear loss of hair follicles and significant muscle atrophy across entire treatment site.

Histopathology confirmed the absence of melanoma in treatment areas in all samples evaluated. Evidence of scar in all treatment groups was only mild (**Figure 6**, **7**, **S1-4**, **Table S1**). Dermal fibrosis, width of fibrosis, hair follicle loss, and cutaneous trunci muscle atrophy/loss were on average mild or mostly mild. Notably, dermal fibrosis was significantly greater (*p=0.0179) after treatment with a high dose of cryoablation (90s) relative to a low dose (45s) (**Figures 6**, **S1-2**, **Table S1**). However, there were no statistically significant differences between the remainder of the treatment groups for lesion scores. Inflammation was mostly absent in most samples, regardless of treatment and did not differ significantly. The epidermis was intact in all samples and erosions and ulcers were not present.

## Discussion

The B16-F10 murine melanoma model was the first used to demonstrate the ability of NPS therapy to permanently eliminate intradermal melanomas (14, 15, 28). It was discovered quite early that the minimum electric field strength required was on the order of 20 kV/cm and that the most likely mechanism involved the formation of pores in lipid membranes (29, 30). The additional discovery that only about 400 mV is required across a lipid membrane to electropermeabilize it (31), suggested that the approximate size of the NPS target must be on the order of 0.2 um, the size of smaller intracellular organelles such as mitochondria. A single NPS pulse has little effect on the tumor but as the pulse number increases, the electropermeabilization effect becomes more evident (**Figure 2**). However, this response does not depend on any significant temperature increase (32, 33) which indicates the cell death mechanism induced by NPS is of a non-thermal nature.

NPS has also demonstrated high efficacy in the treatment of tumors in other murine models of cancer. The tumor elimination rate is typically around 75-100% dependent upon the model and treatment energy used (34). NPS demonstrated 100% efficacy in eliminating 4T1 murine breast cancer in one study (35), in another study a 75% elimination rate was observed after NPS treatment of mouse hepatocellular carcinomas (36) and a 80-90% response rate was noted in the treatment of rat hepatocellular carcinomas (37). Within our laboratory alone, we have shown that an energy of 360 mJ/mm^3^ eliminates between 90-100% of tumors across several murine tumor types, including B16-F10 melanoma (**Figure 2**). Response rates are related to tumor type and size as well as treatment energy. NPS has also typically exhibited an ability to induce an immune response after treatment, likely due to the immunogenic nature of the RCD process triggered by NPS (3, 37–40).

In addition to the high efficacy in treating murine tumors, NPS exhibits similar high levels of efficacy in treating human skin lesions while producing limited damage to the skin itself (8, 41, 42). Histologic examination has shown only a minimal degree of epidermal and dermal inflammation associated with NPS treatments and this was less than typically observed in skin treated with cryoablative and other physical methods of lesion removal. These low levels of inflammation lead to less abnormal collagen deposition resulting in less dermal fibrosis and therefore less permanent scaring (43). Clinical trials utilizing NPS in the treatment of seborrheic keratosis, sebaceous gland hyperplasia, non-genital warts and basal cell carcinoma have all shown successful treatment outcomes (8, 11, 41, 42). Based on the results of these trials the CellFX^®^ device, used to deliver NPS energy, recently attained medical device clearance for the treatment of human benign skin lesions in the USA (FDA 510(k)), Canada (Health Canada) and the EU (CE mark).

While NPS and cryoablation share treatment similarities, the mechanism each uses to destroy cells is quite different. NPS uses ultrashort, high voltage electric pulses that generate transient nanopores in cell and organelle membranes, leading to the initiation of a regulated cell death process in the exposed cells while leaving acellular tissue components unharmed (43, 44). It can also treat tissues with more precise boundaries than thermal-based treatment modalities, ensuring the treatment zone is highly focal to the lesion (3). In contrast, the cryoablation mechanism of cell death involves the quick drastic cooling of the tissue to -40°C which leads to the formation of ice (26, 45). Ice formation causes immediate cell shrinkage and damage to intracellular proteins and membranes. Over time the continued exposure to extremely low temperatures causes thrombosis, tissue hypoxia and eventual necrosis. Cryo exposure also causes cell death indirectly, as the formation of ice within tissues can destroy supporting structural tissue and vasculature that is required for the survival of cells. Vascular endothelial cells can be significantly damaged, and as the tissues gradually thaw, reperfusion draws in platelets that can cause significant clotting and blockage of blood vessels (46–48). This ischemic outcome serves to starve the treated tissue of needed blood supply. The ischemia can also cause hyperemia, erythema, and edema through the production of molecules that cause vasodilation and inflammation (47, 49). While these effects are critical to the mechanism of cryo-induced cell death they also have the potential to significantly damage surrounding tissues due to thermal diffusion, particularly if longer exposure times and multiple cycles are being utilized for treatment.

One of the biggest potential drawbacks to the use of cryosurgical techniques, is that regimens aggressive enough to completely eliminate tumors without recurrence are also highly damaging to other tissues. When cryotherapy is used to ablate cancerous lesions in the clinic it requires the use of multiple cycles and longer freeze times, which increases the likelihood of scarring and damage to underlying structures and peripheral tissues (50, 51). This has kept cryotherapy from being a recommended first line therapy for most malignant lesions and tumors and only remains an option when surgical excision is not (24). The current strategy for elimination of cancerous lesions with cryo therapies such as non-melanoma skin cancers (NMSCs), prostate, kidney or hepatic lesions (52) is typically to overtreat and extend the margins into the periphery to ensure complete elimination of all fast growing malignant cells to prevent reccurrence or even metastasis (52). Obviously, the complete elimination of all tumor cells is imperative, and the primary endpoint of any treatment used to eliminate a malignant lesion. However, minimizing the destruction of normal cells and tissues is also vital and an obvious objective in any clinical trial. Thus, one of the largest potential benefits of NPS treatment over cryoablation therapies is its high rate of tumor clearance at a dose that demonstrates very minimal damage to surrounding tissues.

Additionally, when cryoablative treatments are used to treat melanomas, they are typically used in combination with immunotherapies, other surgical procedures and/or to debulk non-surgically accessible metastatic lesions (53, 54). Trials are currently being conducted to investigate the use of therapies that combine immune adjuvants and/or immune checkpoint blockade with cryotherapy to treat melanoma and other aggressive cancers (55–57). These studies are intended to harness the immune response that is induced by the release of antigens after treatment and direct it towards an adaptive CD8+ memory response that has the ability to target and destroy tumor cells left over after the primary mechanism of cell death has ceased (57). The potential for abscopal effects that may target metastatic sites is also being investigated (58).

Although we only examined the single-agent efficacy of NPS on primary tumor elimination in this study, previous published studies have documented the ability of NPS to inhibit both the growth of a tumor cell rechallenge and prevent metastasis in a CD8-dependent manner (13, 15, 17, 18, 34, 38). The RCD process induced by NPS is likely responsible for priming this CD8^+^ T cell- mediated immune response (44). The combination of NPS with immune adjuvants appears to have an additive effect that boosts treatment efficacy and prevents the growth of a tumor cell rechallenge as evidenced in studies conducted within our laboratory. In the future, we may plan studies to compare the immune responses induced by NPS with those induced by cryoablation.

## Conclusion

NPS displayed superior efficacy over cryoablation with negligible impact to the skin tissue in our side-by-side preclinical comparison. Although NPS has not yet been used in the human clinic to treat aggressive malignant tumors such as melanoma, it has displayed a high rate of efficacy in the treatment of murine tumor types that are typically difficult to kill, without reoccurrence. NPS shares many of the features that make cryotherapies attractive, such as the ability to target hard-to-access lesions and tumors that are untreatable with surgical means, without the associated thermal tissue damage characteristic of cryotherapy.

## Data availability statement

The original contributions presented in the study are included in the article/**Supplementary Material**. Further inquiries can be directed to the corresponding authors.

## Ethics statement

The animal study was reviewed and approved by Pulse Biosciences IACUC.

## Author contributions

AM, BF, CN, KVR, DG and RN conducted all the animal treatments. KL evaluated and scored the histological sections. AM wrote the first draft of the manuscript and RN revised to incorporate the reviewer’s suggestions. All authors contributed to the article and approved the submitted version.

## Supplementary material

Supplementary Figure 1 Representative composite image based on average severity scoring for Cryo (45s) condition, shown at 10X and 40X magnification. Severity scoring for all metrics of tissue damage were as follows: Dermal Fibrosis = 1; Lesion Width = 1; Follicle Loss = 1; Muscle Atrophy = 1; Inflammation = 1.

Supplementary Figure 2 Representative composite image based on average severity scoring for Cryo (90s) condition, shown at 10X and 40X magnification. Severity scoring for all metrics of tissue damage were as follows: Dermal Fibrosis = 1; Lesion Width = 2; Follicle Loss = 2; Muscle Atrophy = 2; Inflammation = 0.

Supplementary Figure 3 Representative composite image based on average severity scoring for NPS (180 mJ/mm3) condition, shown at 10X and 40X magnification. Severity scoring for all metrics of tissue damage were as follows: Dermal Fibrosis = 1; Lesion Width = 1; Follicle Loss = 1; Muscle Atrophy = 1; Inflammation = 1.

Supplementary Figure 4 Representative composite image based on average severity scoring for NPS (360 mJ/mm3) condition, shown at 10X and 40X magnification. Severity scoring for all metrics of tissue damage were as follows: Dermal Fibrosis = 1; Lesion Width = 1; Follicle Loss = 1; Muscle Atrophy = 2; Inflammation = 0.
