# Supplementary figures and images for: Nano-pulse stimulation™ therapy (NPS™) is superior to cryoablation in clearing murine melanoma tumors
Source: Front Oncol. 2023 Feb 8;12:948472. doi: 10.3389/fonc.2022.948472 (PMC9945337; doi:10.3389/fonc.2022.948472)

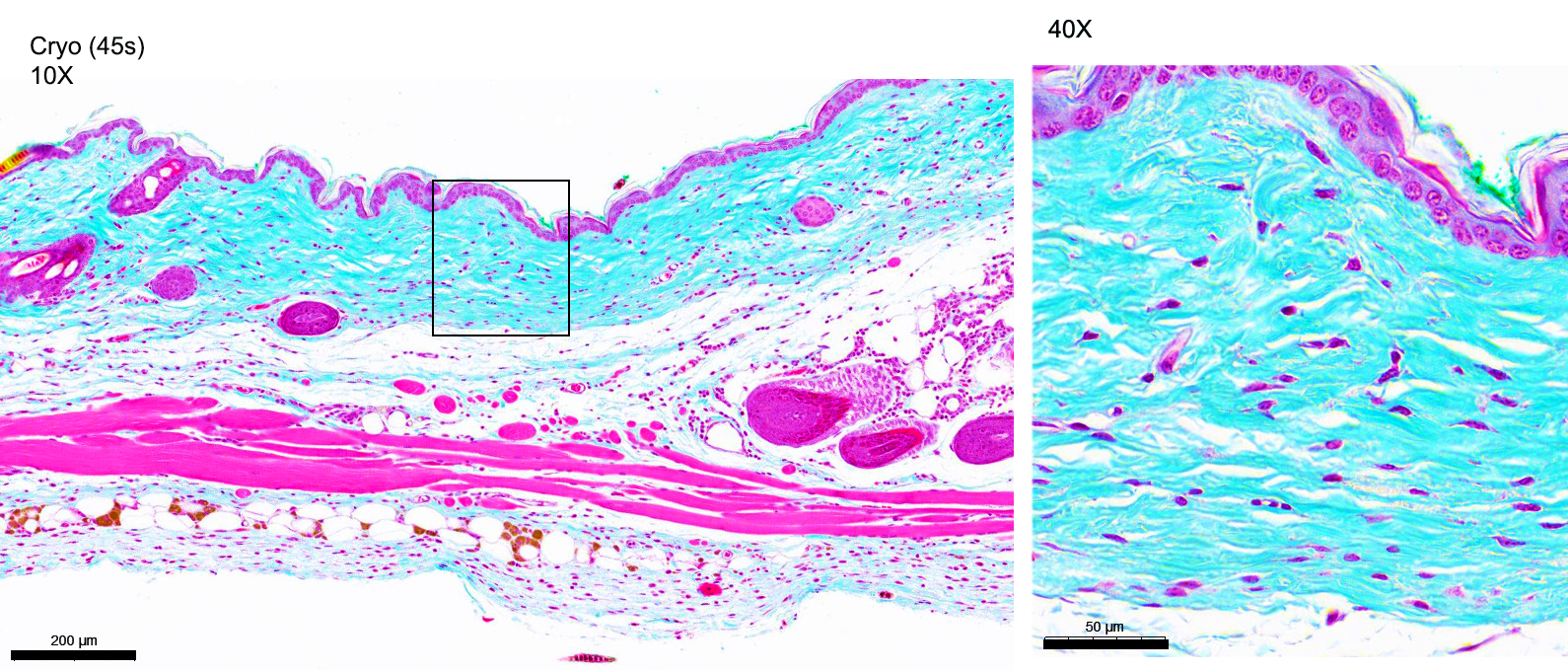

Supplement: Supplementary Figure 1 — Representative composite image based on average severity scoring for Cryo (45s) condition, shown at 10X and 40X magnification. Severity scoring for all metrics of tissue damage were as follows: Dermal Fibrosis = 1; Lesion Width = 1; Follicle Loss = 1; Muscle Atrophy = 1; Inflammation = 1. [file Image_1.tif]

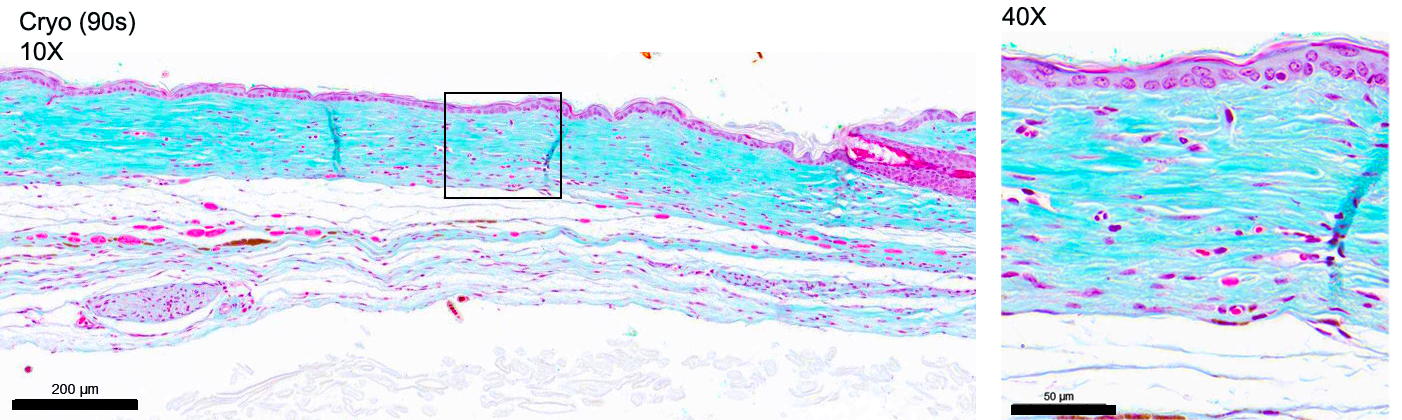

Supplement: Supplementary Figure 2 — Representative composite image based on average severity scoring for Cryo (90s) condition, shown at 10X and 40X magnification. Severity scoring for all metrics of tissue damage were as follows: Dermal Fibrosis = 1; Lesion Width = 2; Follicle Loss = 2; Muscle Atrophy = 2; Inflammation = 0. [file Image_2.tif]

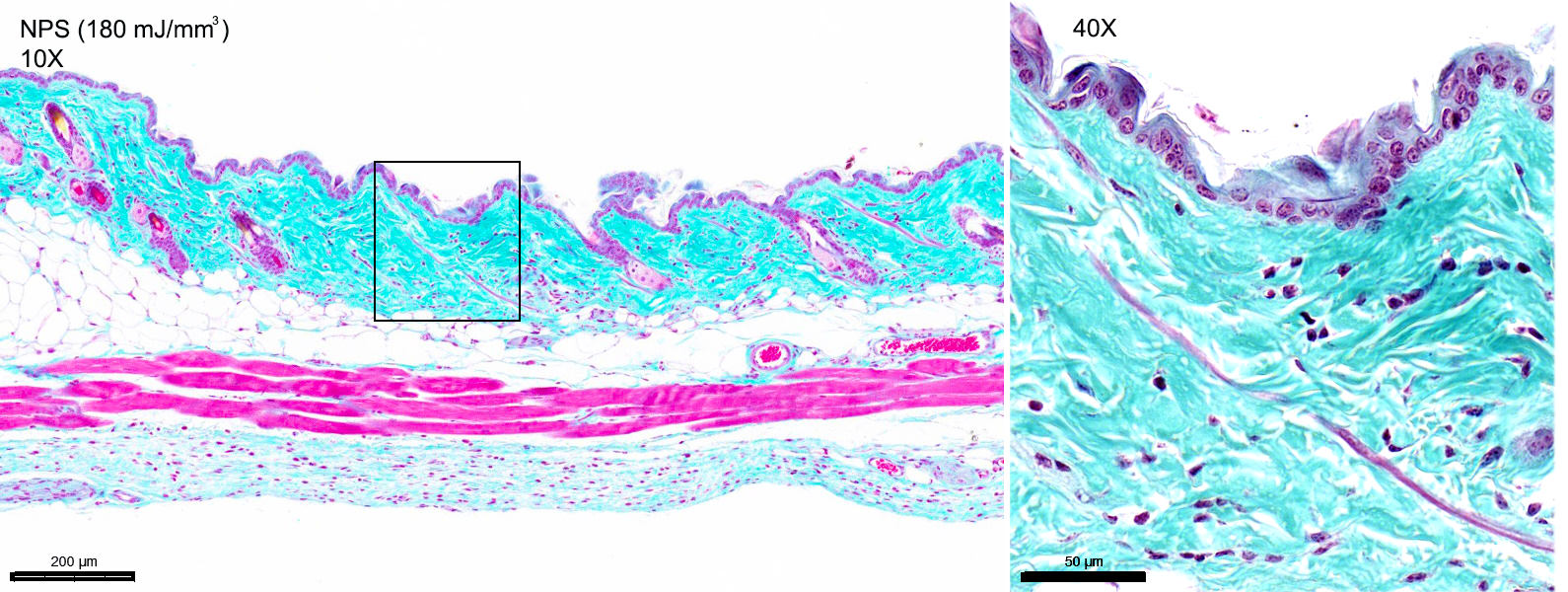

Supplement: Supplementary Figure 3 — Representative composite image based on average severity scoring for NPS (180 mJ/mm3) condition, shown at 10X and 40X magnification. Severity scoring for all metrics of tissue damage were as follows: Dermal Fibrosis = 1; Lesion Width = 1; Follicle Loss = 1; Muscle Atrophy = 1; Inflammation = 1. [file Image_3.tif]

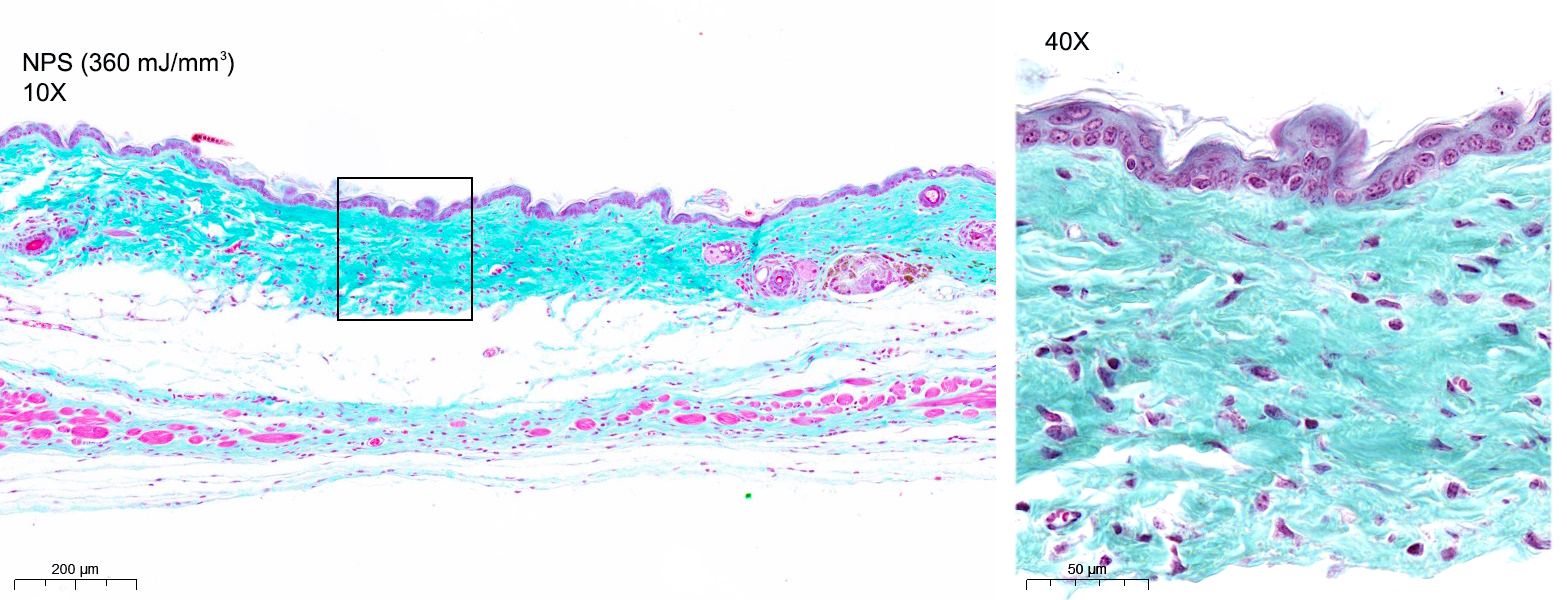

Supplement: Supplementary Figure 4 — Representative composite image based on average severity scoring for NPS (360 mJ/mm3) condition, shown at 10X and 40X magnification. Severity scoring for all metrics of tissue damage were as follows: Dermal Fibrosis = 1; Lesion Width = 1; Follicle Loss = 1; Muscle Atrophy = 2; Inflammation = 0. [file Image_4.tif]
