# Supplementary material for: Nano-pulse stimulation™ therapy (NPS™) is superior to cryoablation in clearing murine melanoma tumors
Source: Front Oncol. 2023 Feb 8;12:948472. doi: 10.3389/fonc.2022.948472 (PMC9945337; doi:10.3389/fonc.2022.948472)
Supplement: Supplementary file 5 [file Table_1.docx]

**Supplementary Table 1.** Severity Scores For Representative Images

| **Condition** | **Dermal Fibrosis** | **Lesion**  **Width** | **Follicle**  **Loss** | **Muscle**  **Atrophy** | **Inflammation** |
| --- | --- | --- | --- | --- | --- |
| **Cryo (45s)** | 1 | 1 | 1 | 1 | 1 |
| **Cryo (90s)** | 1 | 2 | 2 | 2 | 0 |
| **NPS (180 mJ/mm^3^)** | 1 | 1 | 1 | 1 | 1 |
| **NPS (360 mJ/mm^3^)** | 1 | 1 | 1 | 2 | 0 |
